# Neuropsychiatric changes following striatal stroke– results from the observational PostPsyDis study

**DOI:** 10.1186/s42466-025-00390-3

**Published:** 2025-05-12

**Authors:** Anna Kufner, Ana Sofía Ríos, Benjamin Winter, Uchralt Temuulen, Ahmed Khalil, Ulrike Grittner, Johanna Schöner, Asli Akdeniz, Ulrike Lachmann, Golo Kronenberg, Arno Villringer, Karen Gertz, Matthias Endres

**Affiliations:** 1 Klinik für Neurologie mit Experimenteller Neurologie, Charité– Universitätsmedizin Berlin, corporate member of Freie Universität Berlin and Humboldt-Universität zu Berlin, 12203 Berlin, Germany; 2 Center for Stroke Research Berlin (CSB), Charité - Universitätsmedizin Berlin, corporate member of Freie Universität Berlin and Humboldt-Universität zu Berlin, 12203 Berlin, Germany; 3 Berlin Institute of Health at Charité– Universitätsmedizin Berlin, 10117 Berlin, Germany; 4 NeuroCure Clinical Research Center (NCRC), Charité - Universitätsmedizin Berlin, corporate member of Freie Universität Berlin and Humboldt-Universität zu Berlin, NeuroCure Cluster of Excellence, 10117 Berlin, Germany; 5 Department of Neurology, Alexianer St. Josefs-Krankenhaus Potsdam, Potsdam, Germany; 6 Medizinische Hochschule Brandenburg - Theodor Fontane, Neuruppin, Germany; 7 Experimental and Clinical Research Center, Charité - Universitätsmedizin Berlin, Corporate Member of Freie Universität Berlin and Humboldt-Universität Zu Berlin, Berlin, Germany; 8 Institut für Biometrie und klinische Epidemiologie, Charité– Universitätsmedizin Berlin, corporate member of Freie Universität Berlin and Humboldt-Universität zu Berlin, 10117 Berlin, Germany; 9 Klinik für Psychiatrie und Psychotherapie, Charité– Universitätsmedizin Berlin, corporate member of Freie Universität Berlin and Humboldt-Universität zu Berlin, 12203 Berlin, Germany; 10 Department of Neurology, Max Planck Institute for Human Cognitive and Brain Sciences, Leipzig, Germany; 11 German Centre for Cardiovascular Research (Deutsches Zentrum für Herz-Kreislauf- Forschung, DZHK), Partner Site Berlin, 10117 Berlin, Germany; 12 German Center for Neurodegenerative Diseases (Deutsches Zentrum für Neurodegenerative Erkrankungen, DZNE), Partner Site Berlin, 10117 Berlin, Germany; 13 German Center for Mental Health (DZPG), Partner Site Berlin, 10117 Berlin, Germany

**Keywords:** Post-stroke depression, Post-traumatic stress disorder (PTSD), Striatal lesions, Neuropsychiatric outcomes, Ischemic stroke

## Abstract

**Background:**

Ischemic stroke can lead to neuropsychiatric sequelae such as depression and post-traumatic stress disorder (PTSD), resulting in poorer functional outcomes. The POST-stroke PSYchological DIStress PostPsyDis; NCT01187342) study aimed to investigate whether ischemic lesions in the striatum increase the risk of depression and PTSD after stroke.

**Methods:**

This monocenter, observational, case-control study included 84 ischemic stroke patients with striatal (*n* = 54) and non-striatal ischemic brain lesions (*n* = 30). Primary study endpoints included symptoms of depression (assessed via the Geriatric Depression Scale; GDS-30) and PTSD (assessed via the Posttraumatic Symptom Scale; PTSS-10) 90 days post-stroke. A normative functional connectome was used to obtain a measure of striatal connectivity to the rest of the brain (“striatal network”). Network damage scores were used to estimate damage of each lesion to the striatal network.

**Results:**

Patients with striatal lesions had higher GDS-30 scores at 90 days post-stroke (median 5.6 vs. 3.0; Cohen’s d = 0.39; *p* = 0.057), indicating a small to moderate effect. However, no meaningful group differences were observed in the incidence of depression or PTSD. In multivariable regression analyses, striatal infarction had an adjusted beta coefficient (β) of 1.9 (95%CI 0.19–3.7; *p* = 0.076) for GDS-10 and 1.8 (95%CI -1.9–5.5; *p* = 0.25) for PTSS-10 scores after 90 days. Only female sex was independently associated with PTSD severity (adjusted β = 5.1, 95% CI 1.3–8.8; *p* = 0.008). Analyzing lesion connectivity to the striatal network did not change these findings.

**Conclusions:**

Taken together, the PostPsyDis study suggests a high rate of psychiatric morbidity in stroke patients. Moreover, the study suggests increased neuropsychiatric symptoms in patients with striatal lesions. There is a clear need for larger studies to investigate the role of the striatum in post-stroke neuropsychiatric disorders.

**Trial registration:**

ClinicalTrials.gov (NCT01187342) Registered 23 August 2009, https://clinicaltrials.gov/study/NCT01187342.

**Supplementary Information:**

The online version contains supplementary material available at 10.1186/s42466-025-00390-3.

## Background

Psychosocial stress is a well-established risk factor for cardiovascular disease, including myocardial infarction and stroke. However, the relationship between cardiovascular diseases and stress appears to be bidirectional. Direct tissue damage from cardiovascular events may trigger symptoms of psychological distress by disrupting neurobiological systems involved in emotional regulation and stress response [1, 2]. Neuropsychiatric disorders such as depression and post-traumatic stress disorder (PTSD) are common approximately 12 months after ischemic stroke (30% and 15%, respectively) and significantly impair recovery and quality of life [3, 4, 5. Although younger age, female sex and maladaptive coping strategies have been identified as risk factors [5, 6, 7, 8], the mechanisms underlying neuropsychiatric symptoms after stroke remain poorly understood.

Preclinical studies have shown that ischemic lesions to the striatum can lead to secondary neurodegeneration in key midbrain structures, disrupting the mesolimbic reward system and stress-related networks [9, 10]. These disruptions correlate with behavioural changes in rodent models, such as increased anxiety and hyperactivity [1, 2], suggesting that striatal lesions impair stress and reward systems through disruption of striato-nigral and mesolimbic pathways [1, 11].

Based on these preclinical findings, the prospective observational study POST-stroke PSYchological DIStress (PostPsyDis) was initiated to investigate whether patients with striatal infarcts have higher rates of depression and PTSD symptoms than patients without striatal involvement. We included an additional post-hoc, exploratory analysis in which we performed lesion connectivity analyses based on a normative functional connectome to determine whether damage to striatal networks rather than direct lesion to the striatum increase the risk of depression and PTSD after stroke.

## Methods

### Study design and patient population

The PostPsyDis study (ClinicalTrials.gov NCT01187342) was a monocenter, observational, case-control study conducted at Charité-Universitätsmedizin Berlin from October 2009 to December 2013. Patients were recruited consecutively during acute hospital admission at Campus Benjamin Franklin Charité. Eligible patients were identified through stroke unit records and screened within 72 h of admission. The study’s primary objective was to investigate whether ischemic lesions to the striatum increased the incidence of post-stroke affective symptoms, specifically depression and PTSD. A two-group design was employed, categorizing patients into striatal lesion (defined as lesions to the caudate nucleus, pallidum, putamen, and/or nucleus accumbens) and non-striatal lesion groups. Main inclusion criteria included: acute ischemic stroke within the middle cerebral artery (MCA) or anterior choroidal artery (AchA) territories, with or without striato-capsular involvement. To adjust for the effect of stroke severity (National Institute of Stroke Scale; NIHSS) on primary study endpoints, inclusion criteria for lesion volumes differed between groups. For MCA infarcts with striatal involvement, lesion size had to be ≥125 mm³ (0.125 mL) and could include either lacunar striato-capsular or territorial cortical infarcts. For non-striatal MCA infarcts, lesion size could range from 10 to 100 cm³ (10–100 mL). AchA infarcts required involvement of the posterior capsule (disruption of nigrostriatal pathways), putamen, and/or pallidum [12]. For a detailed list of all study inclusion and exclusion criteria, refer to Supplementary Table 1. All participants provided written informed consent under the supervision of a witness. The study adhered to the Declaration of Helsinki and was approved by the local ethics committee in Berlin.

An a priori sample size calculation estimated that 88 participants (44 per group) were needed to detect group differences in post-stroke depression and PTSD, assuming a medium effect size (w = 0.3), α = 0.05, power = 0.80, and equal group allocation. This estimate was based on Cohen’s conventional effect size guidelines and literature suggesting moderate associations between lesion location and post-stroke affective outcomes [12, 13, 14]. To account for potential dropouts and enable secondary analyses, the target sample size was increased to 100. However, due to the departure of key study personnel, recruitment ended prematurely, and the final sample fell short of this target. Despite the reduced sample size, all data collection and analyses were conducted according to the original protocol.

### Clinical assessment and primary study endpoints

Patients were assessed at admission (T1), day 1 (T2), at 3 months (T3, inpatient), and 12 months post-stroke (T4, telephone). All patients completed follow-up assessments at both T3 and T4. Neuropsychiatric assessments measured depressive symptoms using the Geriatric Depression Scale (GDS-30). Depression was defined by a score ≥ 10 on the GDS-30. PTSD symptoms were assessed using the Post-Traumatic Stress Syndrome 10-Question Inventory (PTSS-10), with scores ≥ 19 reflecting presence of moderate to severe symptoms of PTSD. Cognitive function was evaluated with the Montreal Cognitive Assessment (MoCA) at T3 and the Telephone Interview for Cognitive Status– Modified (TICS-M) at T4. Quality of life was assessed using the Short Form (SF-12) Health Survey, which provides Physical (PCS) and Mental Component Summary (MCS) scores. These scores are normed to a mean of 50 (standard deviation [SD] ± 10), with higher scores reflecting better health status.

The primary endpoints of the PostPsyDis study were the severity of depressive symptoms (GDS-30) and PTSD symptoms (PTSS-10) assessed 3 months post-stroke (T3) in relation to ischemic lesion location within the striatum. The primary hypothesis was that patients with striatal ischemic lesions would exhibit more severe depressive and PTSD symptoms. Secondary endpoints included the impact of striatal lesions on quality of life (SF-12) and cognitive function evaluated at T3.

Cohen’s d effect sizes and retrospective power analyses were conducted for GDS-30 and PTSS-10 outcomes (α = 0.05). Effect sizes were classified as small (d = 0.2), medium (d = 0.5), or large (d = 0.8), with adequate power defined as > 80%. For GDS-30, a small effect size (d = 0.31, 95% CI: -0.174 to 0.794) yielded 44.5% power, PTSS-10 showed a negligible effect size (d = 0.014, 95% CI: -0.475 to 0.503), with 5.1% power.

### Imaging processing

Patients underwent a standardized magnetic resonance imaging (MRI) protocol on a 3-Tesla scanner (Tim Trio; Siemens, Erlangen, Germany). Lesions were manually delineated on diffusion-weighted imaging (DWI; TE = 93.1 ms, TR = 7600 ms, FOV = 230 mm, matrix = 192 × 192, 2.5-mm section thickness with no intersection gap) slices using MRIcron (https://www.nitrc.org/projects/mricron), blinded to clinical data and verified independently by at least two independent neurologists. Lesion masks were co-registered to brain-extracted b0 after skull stripping using the Brain Extraction Tool (FSL) and normalized to Montreal Neurological Institute (MNI152 atlas, 1 × 1 × 1 mm) space through linear and non-linear registrations using Advanced Normalization Tools in Python (ANTsPy).

### Lesion symptom mapping

Lesion symptom mapping (LSM) analyses were conducted using NiiStat (https://www.nitrc.org/projects/niistat/) to examine associations between brain lesions and the development of depression and PTSD. Clinical data were available for 70 participants (depression) and 68 (PTSD) at 12 months post-stroke—a time point selected based on previous studies [15, 16]. Analysis parameters included a minimum lesion overlap of 3 participants (5% of the sample [17], regression of lesion volume to reduce confounding, [18] and 5,000 permutations for voxelwise inference with familywise error correction. Statistical significance was defined as *p* < 0.05. Given the main study hypothesis that ischemic damage to the striatum contributes to post-stroke neuropsychiatric symptoms, we also conducted a region-of-interest (ROI)–based LSM analysis focused on the the striatum only for both PSD and PTSD.

### Striatal network connectivity analysis

To assess whether lesion connectivity to the striatal network influenced the development of the primary study endpoints, a striatal mask stemming from caudate, putamen, pallidum and nucleus accumbens from the Harvard Oxford Subcortical Structural Atlas [19, 20] was used as seed to calculate functional connectivity from a normative connectome with rs-fMRI data from 1,000 healthy subjects using the lead-mapper tool from the Lead-DBS toolbox (lead-dbs.org) [21]. For each patient, a “striatal network damage score” was calculated by summing voxel intensities within the striatal network overlapping with the lesion, and adjusted for lesion volume, as described previously [22]. The striatal network damage scores were then included as variables in a linear mixed-model analysis.

## Results

### Cohort description

A total of 84 patients were enrolled in the study (54 with striatal and 30 with non-striatal lesions). Mean age was 66.8 years (SD 12.4), 34 were female (40.5%), median NIHSS was 2 (IQR 2–4). Basic patient demographics in terms of sex, age, and cerebrovascular risk profiles were similar between groups (Table 1). According to the study design, patients with non-striatal lesions had larger baseline lesion volumes (median 9.7 mL vs. 1.9 mL, *p* < 0.001), while admission NIHSS scores were comparable between groups (striatal: median NIHSS 2.5, IQR 1–4; non-striatal: median NIHSS 1, IQR 0–4, *p* = 0.11). Stroke-specific scores including functional dependence, and activities of daily life assessed at T3 also did not differ between groups. Four patients were treated with antidepressants at the time of enrollment (3 in striatal and 1 in non-striatal lesion group; Table 1).

**Table 1:** Patient characteristics and demographics

	Total cohort *N* = 84	Striatal *N* = 54	Non-striatal *N* = 30
Age, mean (± SD)	66.8 (12.4)	67.1 (13.2)	66.1 (10.8)
Sex, female, n (%)	34 (40.5)	23 (42.6)	11 (36.7)
Hypertension, n (%)	55 (65.5)	34 (62.9)	21 (70)
Diabetes, n (%)	16 (19.1)	10 (18.5)	6 (20.0)
Hyperlipidemia, n (%)	36 (42.9)	21 (38.9)	15 (50.0)
Atrial fibrillation, n (%)	26 (30.9)	17 (31.5)	9 (30.0)
History of smoking, n (%)	19 (22.6)	12 (22.2)	7 (23.3)
TOAST Classification (Adams 1993)			
Large artery atherosclerosis, n (%)	45 (53.6)	25 (46.3)	20 (66.7)
Cardioembolic stroke, n (%)	26 (30.9)	17 (31.5)	9 (30.0)
Small vessel occlusion, n (%)	7 (8.3)	6 (11.1)	1 (3.3)
Other, n (%)	2 (2.4)	2 (3.7)	0 (0)
Unknown, n (%)	4 (4.8)	4 (7.4)	0 (0)
NIHSS at admission, median (IQR)	2 (1–4)	2.5 (1–4)	1 (0–4)
ARWMC score, median (IQR)	5 (2-9.5)	5 (2–9)	4 (2–10)
DWI lesion volume at baseline, median (IQR)	4.8 (1.0-13.5)	1.9 (0.6–7.1)	9.7 (5.1–25.1)
Thrombolysis, n (%)	33 (39.3)	21 (38.9)	12 (40)
Modified Rankin Score T3, median (IQR)	1 (1–2)	1 (1–2)	1 (0–2)
Barthel Index T3, median (IQR)	100 (100–100)	100 (100–100)	100 (100–100)
Medication with antidepressants T2, n (%)	4 (4.8)	3 (5.6)	1 (3.3)
Medication with antidepressants at any timepoint (T2, T3, T4), n (%)	7 (8.3)	6 (11.1)	1 (3.3)

### Main study results

At T3, the median GDS-30 score for the cohort was 5 (IQR 2–8), with 17.9% of patients with a GDS-30 ≥ 10. At T4, 36.9% (*N* = 31) had a GDS-30 ≥ 10. At T3, the median PTSS-10 score was 8 (IQR 3–14), with 15.5% of patients experiencing moderate to severe PTSD symptoms (PTSS-10 ≥ 19). At T4, 34.5% (*N* = 29) had a PTSS-10 ≥ 19. GDS-30 and PTSS-10 scores were correlated at both T3 and T4 (Pearson’s correlation: 0.68 at T3, 0.80 at T4, *p* < 0.001).

Patients with striatal infarction had higher median GDS-30 scores at T3 (5.6 [IQR 3–9] *versus* 3 [IQR 2–6]), but the effect was only small to moderate indicated by a Cohen’s d of 0.39 (*p* = 0.057; Table 2**)**. There were no group differences in depression incidence, PTSD incidence, or PTSS-10 severity at T3. Secondary outcomes, including health-related quality of life and cognitive function, did not differ between groups (Table 2). In a sub-group analysis including only patients with lesions restricted solely to the striatum (*n* = 18) in comparison to patients with non-striatal lesions, there were no meaningful differences in terms of the primary study endpoints assessed at T3 (Supplementary Table 2). Three patients within the striatal lesion group were newly treated with antidepressants within one year of the index event. No patients in the non-striatal group were newly treated with antidepressants.

**Table 2:** Assessment of primary and secondary outcome parameters based on lesion location assessed at 90 days post-stroke. *Effect sizes are reported as Cohen’s d for continuous variables and odds ratios (OR) with 95% confidence intervals for binary variables

		Striatal (*N* = 54)	Non-striatal (*N* = 30)	*p*-value	Effect Size*	95% CI
**Primary study endpoints**	GDS-30 total score, median (IQR)	5.9 (3–9)	3 (2–6)	**0.057**	0.39	−0.07–0.83
	Depression yes/no, n (%)	12 (22.2)	3 (10)	0.16	0.39	0.1–1.5
	PTSS-10 total score, median (IQR)	10 (2–16)	6 (3–10)	0.25	0.19	−0.26 to 0.64
	PTSD yes/no, n (%)	9 (16.7)	4 (13.3)	0.17	0.77	0.22–2.7
**Secondary study endpoints**	SF-12 PC score, median (IQR)	47.5 (37.3)	53 (41.1–55.9)	0.16	−0.23	−0.76 to 0.29
	SF-12 MCS score, median (IQR)	55.2 (46.0–59.4)	55.2 (52.4–59.3)	0.52	−0.25	−0.76 to 0.26
	MOCA total score, median (IQR)	26 (24–27)	27 (25–29)	0.21	0.13	−0.41 to 0.67

In multivariable regression analysis including age, sex, lesion volume and study group, striatal infarction had an adjusted beta of 1.9 (95% CI 0.19 to 3.7; *p* = 0.076) for higher GDS-30 scores at T3 and an adjusted beta of 1.8 (95%CI -1.9-5.5, *p* = 0.25) for higher PTSS-10 scores at T3. Only female sex was identified as an independent predictor of higher PTSS-10 scores (adjusted beta 5.1, 95% CI 1.3–8.8; *p* = 0.008; Supplementary Table 3). In a sensitivity analysis that included antidepressant medication use at any time (T2–T4) as an additional covariate, the results remained consistent (Supplementary Table 4).

**Fig. 1:**
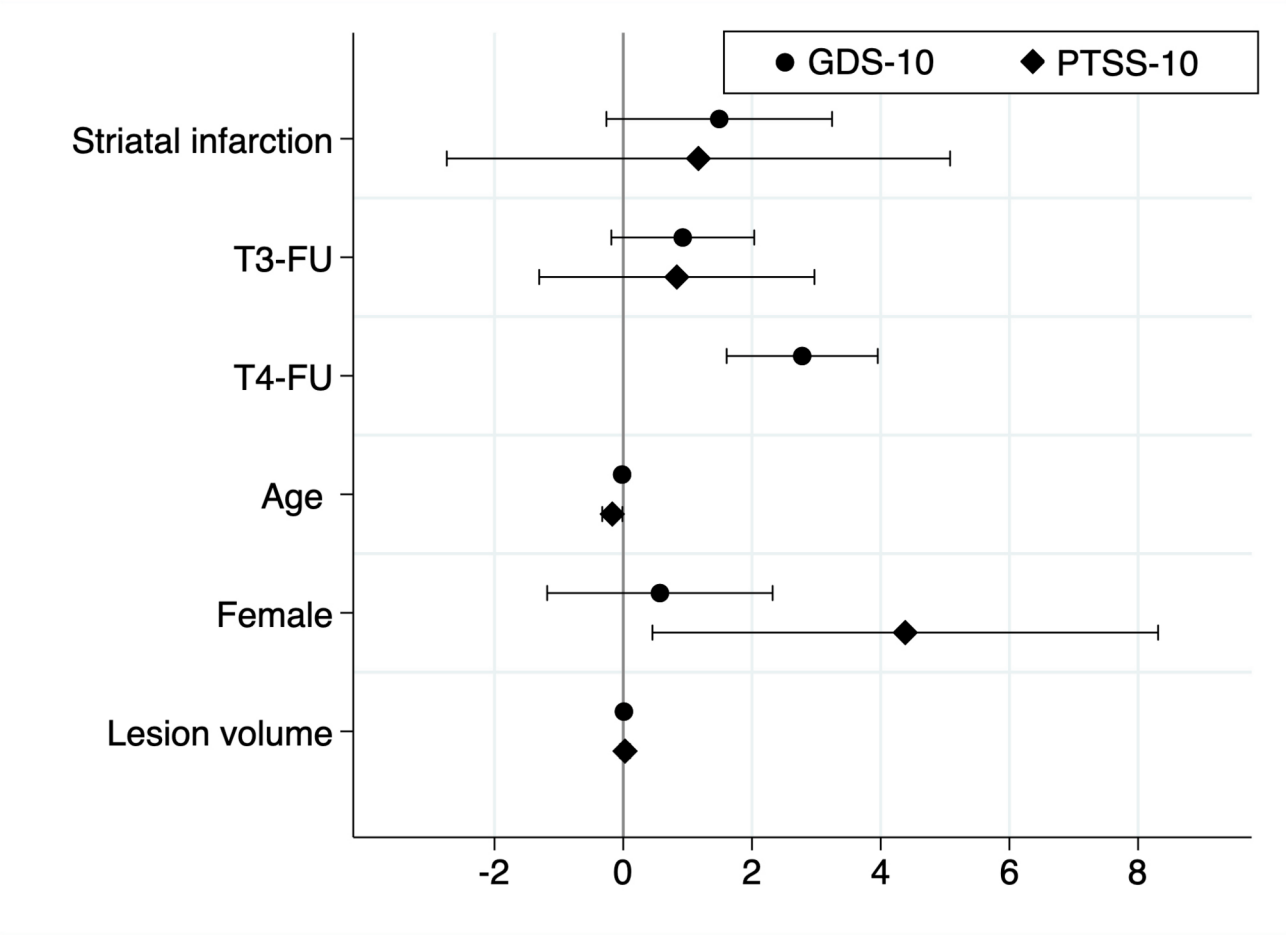
Coefficient plot of linear mixed models for depression (GDS-10) and PTSD (PTSS-10) assessed up to one year following ischemic stroke

In a linear mixed-model analysis for GDS-30 scores across all time-points of assessment, striatal infarction had a coefficient of 1.5 (standard error SE 0.87; *p* = 0.08). For PTSS-10, female sex (coefficient 4.4, SE 1.9; *p* = 0.02) and younger age (coefficient − 0.17, SE 0.08; *p* = 0.03) were significant modifying factors; striatal infarction had a coefficient of 1.2 (SE 1.9; 0 = 0.55; Table 3). Coefficient plots for these models are shown in Fig. 1.

**Table 3:** Linear Mixed-models for each primary outcome variable GDS-30 assessed at T2, T3, and T4 and PTSS-10 (assessed at T3 and T4). All models included subjects as a random effect and study group (striatal *versus* non-striatal), time point of assessment, female sex, age, and lesion volume in millilitres (mL)

Dependent variable: Geriatric Depression Score				
Fixed-effects				
	Coefficient	Std. Error	p-value	95% CI
Time-point of assessment				
T2 (1d	*- reference -*	*- reference -*	-	
T3 (90d)	0.93	0.56	0.100	-0.18–2.0
T4 (12mo)	2.8	0.59	*< 0.001*	1.6–3.9
Striatal infarction	1.5	0.87	*0.089*	-0.2–3.2
Lesion volume (mL)	0.01	0.02	0.588	-0.03–0.05
Female	0.57	0.87	0.509	-1.1–2.3
Age	-0.12	0.03	0.606	-0.09–0.05
**Random-effects**				
	**Estimate**	**Std. Error**		
Subject ID	3.1	0.36		2.4–3.9
**Dependent variable: Post-Traumatic Stress Syndrome 10-Question Inventory**				
**Fixed-effects**				
	**Coefficient**	**Std. Error**	**p-value**	**95% CI**
Time-point of assessment				
T3 (90d)	*- reference -*	*- reference -*	-	
T4 (12mo)	0.83	1.1	0.444	-1.3–2.9
Striatal infarction	1.2	1.9	0.548	-2.6–4.9
Lesion volume (mL)	0.03	0.04	0.476	-0.05–0.10
Female	4.4	1.9	*0.024*	*0.57–8.2*
Age	-0.17	0.08	*0.028*	*-0.32 - -0.02*
**Random-Effects**				
	**Estimate**	**Std. Error**		**95% CI**
Subject ID	6.8	0.84		5.5–8.7

### Lesion symptom mapping and striatal network connectivity

Neither whole-brain LSM analysis nor ROI-based LSM (limited to striatal regions) for depression or PTSD at 12 months post-stroke revealed any significant voxel-wise results. The distribution of lesions included in the LSM analyses is shown in Supplementary Fig. 1.

**Fig. 2:**
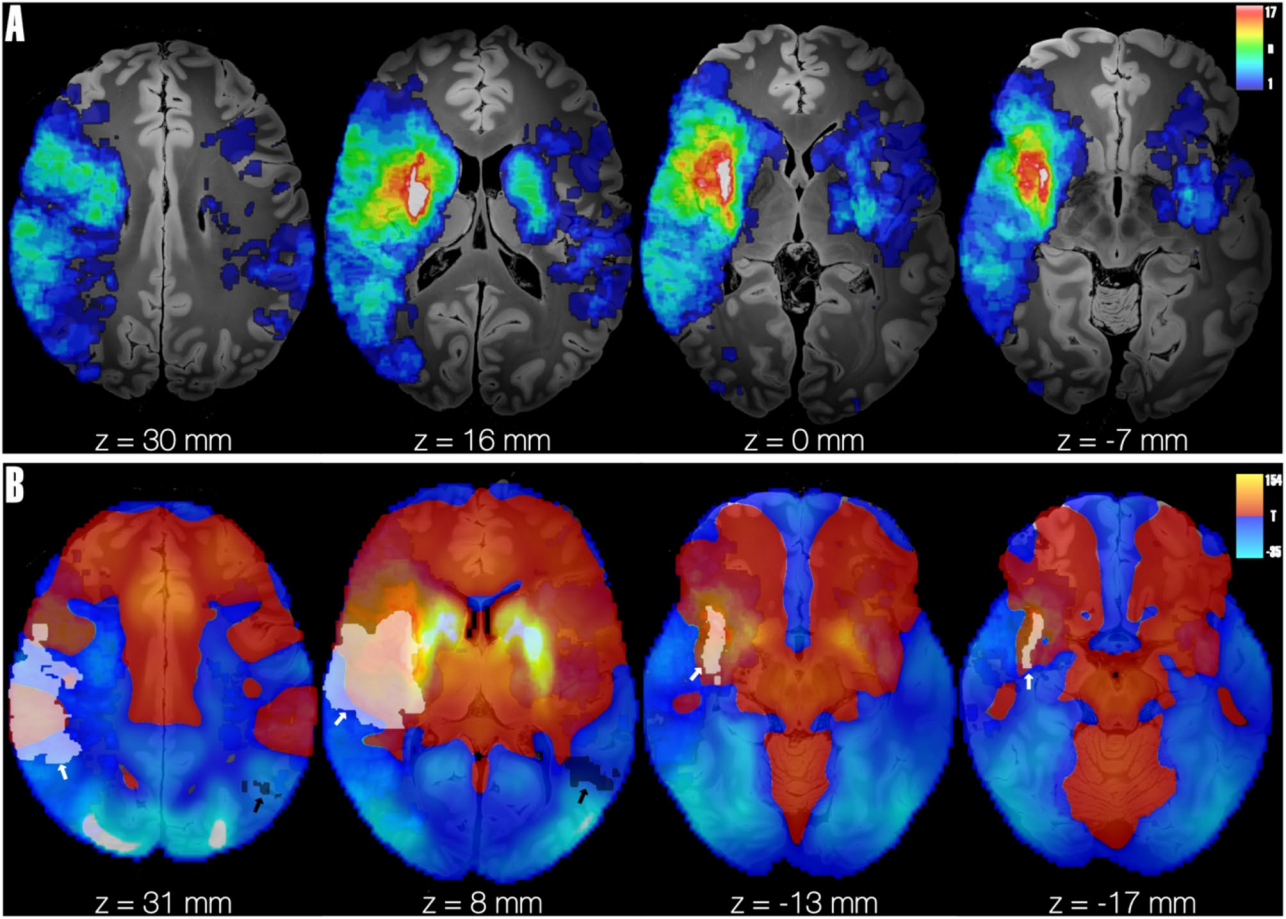
**(A)** Lesion Heatmap. Heatmap of lesions included in the analysis (*n* = 70, maximum overlap = 23) overlaid on axial slices of an ex-vivo MNI space template acquired at 7T with 100 μm resolution [21], cold colours indicate less patients overlap, while warm colours indicate more patients overlap at a given voxel. **(B)** Striatal network. Seed-based connectivity using normative connectome (*n* = 1000 healthy subjects) [14], employing a striatal mask from the Harvard Oxford Subcortical Structural Atlas [12, 13] as the seed. Positive (red-yellow) and negative (blue-light blue) connections based on BOLD signal across the 1000 subjects is shown. Example of two patient lesions from the non-striatal group are shown in white (lesion of patient with GDS-30 and PTSS-10 values above the cut-off for depression and PTSD), pointed by white arrow, and black* (lesion of patient with GDS-30 and PTSS-10 values below clinical cut-off), pointed by black arrow. *Only visible in first two slices

Out of 84 patients, only 70 had quality MRI sequences suitable for lesion delineation and connectivity analyses (Fig. 2). Striatal network damage scores were higher in patients with striatal lesions *versus* patients with non-striatal lesions (median: − 21.7 [IQR − 240.8 to 348.9] *versus* median − 252.9 [IQR − 403.8 to -13.2], *p* < 0.001). Striatal network damage scores showed no differences between patients with GDS-30 ≥ 10 at T3 (*p* = 0.36) or PTSS-10 ≥ 19 at T3 (*p* = 0.85) compared to those with lower scores. The striatal network damage score was not identified as an independent predictor of GDS-10 or PTSS-10 scores in linear mixed-model analysis with an adjusted coefficient of -0.24 (SE 0.6, *p* = 0.696) and − 2.8 (SE 2.2, *p* = 0.208), respectively (Supplementary Table 5).

## Discussion

In this study, we present the main findings of the prospective, observational PostPsyDis study, which included 84 ischemic stroke patients. The primary aim was to investigate whether ischemic lesions to the striatum increase the risk of developing depression and PTSD symptoms 90 days after stroke. While we observed more severe depressive symptoms in patients with striatal lesions, the effect was small to moderate (Cohen’s d of 0.39; Table 2). However, no meaningful group differences were observed for PTSD symptoms, cognitive function, or health-related quality of life.

The rate of post-stroke depression (37% after 12 months) is comparable to the prevalence reported in the literature [3, 23]. Interestingly, the incidence of post-stroke PTSD after 12 months reported in this study (35%) was nearly three times higher than the incidence estimate of 11% at 1 year after stroke or transient ischemic attack reported in an earlier meta-analysis of nine studies [5]. The incidence of PTSD in our sample is significantly higher than that reported in the general German population (5%) [24].

The multivariable regression analysis revealed that striatal infarction was associated with higher GDS-30 and PTSS-10 scores at T3, with adjusted beta coefficients of 1.9 (95% CI 0.19–3.7; *p* = 0.076) and 1.8 (95% CI -1.9–5.5; *p* = 0.25), respectively (Supplementary Table 3). Although these findings were not statistically significant, the positive beta coefficients suggest an increased risk of depression and PTSD symptoms in patients with striatal lesions. The lack of significance may be attributable to the limited sample size and reduced statistical power of the study.

Similar findings were observed in the linear mixed-model analysis, where striatal infarction was associated with a coefficient of 1.5 (SE 0.87; *p* = 0.08; Table 3; Fig. 1) for GDS-30 scores across all time-points, consistent with previous imaging studies linking subcortical structures, including the putamen and pallidum, with depressive symptoms [25, 26]. Interestingly, female sex was independently associated with increased PTSD symptoms in both multivariable linear regression analysis (adjusted beta 5.1, 95% CI 1.3–8.8; *p* = 0.008) and in linear mixed model analysis, underscoring its role as a risk factor for PTSD post-stroke. This aligns with previous and own findings [4, 8].

In the current study, neither lesion volume nor stroke severity, as measured by the NIHSS, appeared to significantly impact the severity of post-stroke neuropsychiatric symptoms (Table 3**)**. While some studies have reported associations between post-stroke depression (PSD) and the extent of neurological deficits [27, 28]—suggesting that depressive symptoms may, in part, reflect psychological responses to new functional limitations—this interpretation remains disputed [23, 29]. Notably, other research has challenged this “adjustment disorder” framework, showing that stroke patients are up to four times more likely to develop depression than orthopedic patients with comparable physical impairments [30, 31]. These findings point to a more complex interplay of biological, functional, and psychosocial factors in the development of post-stroke neuropsychiatric symptoms. It is also worth noting that stroke severity in the current cohort was relatively mild (median NIHSS 2), which may limit the generalizability of these findings to patient cohorts with more severe strokes.

The majority of included infarcts were located in the right hemisphere (Fig. 2A**)**, which is most likely due to the study exclusion criterion of severe aphasia. Although previous studies have suggested an independent association between lesion location in the left internal capsule and post-stroke depression [13], considering discrepant findings across multiple studies on this topic, no definite association between lesion location and post-stroke depression can be established [23]. An additional analysis incorporating each patient’s striatal network damage score caused by the lesion did not reveal any further insights regarding striatal involvement in the primary study endpoints (Supplementary Table 5). Interestingly, striatal involvement has been shown to be associated with post-stroke neuropsychiatric symptoms in several previous lesion symptom mapping studies [25, 29, 32]. In the current study, voxel-based LSM analyses for both depressive symptoms and psychological distress yielded no significant results, neither in the whole-brain analysis nor in the ROI-based analysis limited to the striatum. While the association between striatal lesions and neuropsychiatric outcomes requires further validation, the results of the current studies suggest the importance of considering both lesion characteristics and demographic factors when evaluating post-stroke psychological distress.

This study has several limitations that warrant discussion. First and foremost, the small sample size limits the statistical power to detect differences in the main study outcomes. A post-hoc power analysis revealed limitations in the study’s statistical power, with a power of 0.051 for PTSD and 0.445 for depression, both below the recommended threshold of 0.8 for conclusive results. Given the limited power of this study, including imaging analyses, the results do not provide conclusive evidence. Furthermore, patients with non-striatal lesions had slightly lower NIHSS despite larger lesion volumes (Table 1**)**, which may confound observed trends in neuropsychiatric symptoms [20]. In other words, lesion volume alone does not fully explain severity of neurological deficits or recovery patterns. While analyses were adjusted for lesion volume, this limitation should be considered when interpreting the findings. It is also important to note that the inclusion of patients taking antidepressants at the time of enrolment may have influenced neuropsychiatric symptom profiles; however, the decision not to exclude patients with prior antidepressant use was made to avoid selection bias, given the high prevalence of antidepressant use in the general German population [33]. Additionally, it is important to note that the GDS-30 and PTSS-10 rely on self-reporting, which may be subject to response bias or misinterpretation that cannot be adequately accounted for in our analyses. Lastly, a formal causal framework (e.g., directed acyclic graphs) was not applied to guide covariate selection; instead, adjustment variables were chosen based on established literature and clinical relevance, which may introduce residual confounding. Nevertheless, the main strength of this work is the prospective nature of the PostPsyDis study, which allows for structured and standardised data collection over time. In addition, this is a cohort of well-characterised, homogeneous stroke patients, which has the advantage of strengthening lesion-phenotype associations, especially with respect to neuropsychiatric symptoms after stroke, which has been underexplored in previous studies.

## Conclusions

In conclusion, this pilot study suggests increased depressive and PTSD symptoms in patients with striatal lesions post-stroke, with small-to-moderate effect sizes observed. While these associations did not reach statistical significance, the direction and size of effects indicate a clear need for larger studies to investigate the role of the striatum in post-stroke neuropsychiatric disorders. Lesion connectivity to striatal networks did not improve outcome prediction, but overall, the findings highlight the potential relevance of striatal involvement in post-stroke affective outcomes and the need for validation in larger, adequately powered studies.

## Electronic supplementary material

Below is the link to the electronic supplementary material.

Supplementary Material 1

## Data Availability

Data supporting the results of this study is available upon request from the corresponding author. Open source software were used for the pre-processing and analysis of the data, including: Lead-DBS (https://github.com/netstim/leaddbs*)*, FSL 6.0.6.4 (https://fsl.fmrib.ox.ac.uk/fsl/fslwiki/*)* and ANTsPy (https://github.com/ANTsX/ANTsPy*).*

## Declarations

### Trial registration

ClinicalTrials.gov NCT01187342.

## Abbreviations

AchA: Anterior Choroidal Artery
ANTsPy: Advanced Normalization Tools in Python
BI: Barthel Index
FSL: FMRIB Software Library
GDS-30: Geriatric Depression Scale (30-item)
IQR: Interquartile Range
MCA: Middle Cerebral Artery
MCS: Mental Component Summary (from SF-12)
MoCA: Montreal Cognitive Assessment
MRI: Magnetic Resonance Imaging
NIHSS: National Institute of Stroke Scale
PCS: Physical Component Summary (from SF-12)
PostPsyDis: Post-Stroke Psychological Distress
PTSD: Post-Traumatic Stress Disorder
PTSS-10: Post-Traumatic Stress Syndrome 10-Question Inventory
SD: Standard Deviation
SE: Standard Error
SF-12: Short Form Health Survey (12 items)
TICS-M: Telephone Interview for Cognitive Status– Modified
