# Supplementary material for: Neuropsychiatric changes following striatal stroke– results from the observational PostPsyDis study
Source: Neurol Res Pract. 2025 May 12;7(1):32. doi: 10.1186/s42466-025-00390-3 (PMC12067745; doi:10.1186/s42466-025-00390-3)
Supplement: Supplementary file 1 — Supplementary Material 1 [file 42466_2025_390_MOESM1_ESM.docx]

- **Supplementary Material -**

**Neuropsychiatric Changes Following Striatal Stroke – Results from the Observational PostPsyDis Study**

Anna Kufner, Ana Sofía Ríos, Benjamin Winter, Uchralt Temuulen, Ahmed Khalil, Ulrike Grittner, Johanna Schöner, Asli Akdeniz, Ulrike Lachmann, Golo Kronenberg, Arno Villringer, Karen Gertz, Matthias Endres

| **Inclusion criteria** | **Exclusion criteria** |
| --- | --- |
| - Acute ischemic stroke within the middle cerebral artery (MCA) or anterior choroidal artery (AchA) territories, with or without striato-capsular involvement. - MCA infarcts with striatal involvement:   - Lesion size >125 mm³ (0.125 mL).   - Includes lacunar striato-capsular or territorial cortical infarcts. - Non-striatal MCA infarcts:   - Lesion size between 10–100 cm³ (10–100 mL). - AchA infarcts:   - Required involvement of the posterior capsule, putamen, and/or pallidum. - Additional criteria:   - Age ≥18 years.   - Ability to provide informed consent.   - National Institute of Health Stroke Scale (NIHSS) score ≤20 on admission.   - MRI performed within 24–48 hours of symptom onset | - NIHSS score ≥20. - Clinical instability (e.g., hemodynamic or cardio-respiratory instability, severe infection). - Life expectancy <1 year. - Significant pre-existing brain damage:   - 2 prior strokes.   - Extensive global atrophy.   - Subcortical leukoencephalopathy.   - Brain tumors. - Severe aphasia. - Pre-existing dementia (Mini Mental State Examination [MMSE] score ≤18). - Prior psychiatric conditions (e.g., depression, substance abuse). - Parkinson’s disease (including dopamine agonist use). - MRI contraindications. |

**Supplementary Table 1**: Summary of main inclusion and exclusion criteria for enrollment in the PostPsyDis study.

|  |  | **Only Striatal**  **(N=18)** | **Non-striatal**  **(N=30)** | **p-value** | **Effect Size*** | **95% CI** |
| --- | --- | --- | --- | --- | --- | --- |
| **Primary study endpoints** | GDS-30 total score, median (IQR) | 4.5 (2-8) | 3 (2-6) | 0.40 | 0.30 | -0.29 – 0.88 |
|  | Depression yes/no, n (%) | 4 (22.2) | 3 (10) | 0.24 | 0.39 | 0.08 – 1.9 |
|  | PTSS-10 total score, median (IQR) | 9.5 (2-17) | 6 (3-10) | 0.67 | 0.14 | -0.45 – 0.72 |
|  | PTSD yes/no, n (%) | 4 (22.2) | 4 (13.3) | 0.42 | 0.54 | 0.12 – 2.5 |

**Supplementary Table 2**: Sub-group analysis including only patients with lesions restricted to the striatum (N=18) in comparison to patients with non-striatal lesions (N=30). Assessment of primary outcome parameters based on lesion location assessed at 90 days post-stroke.


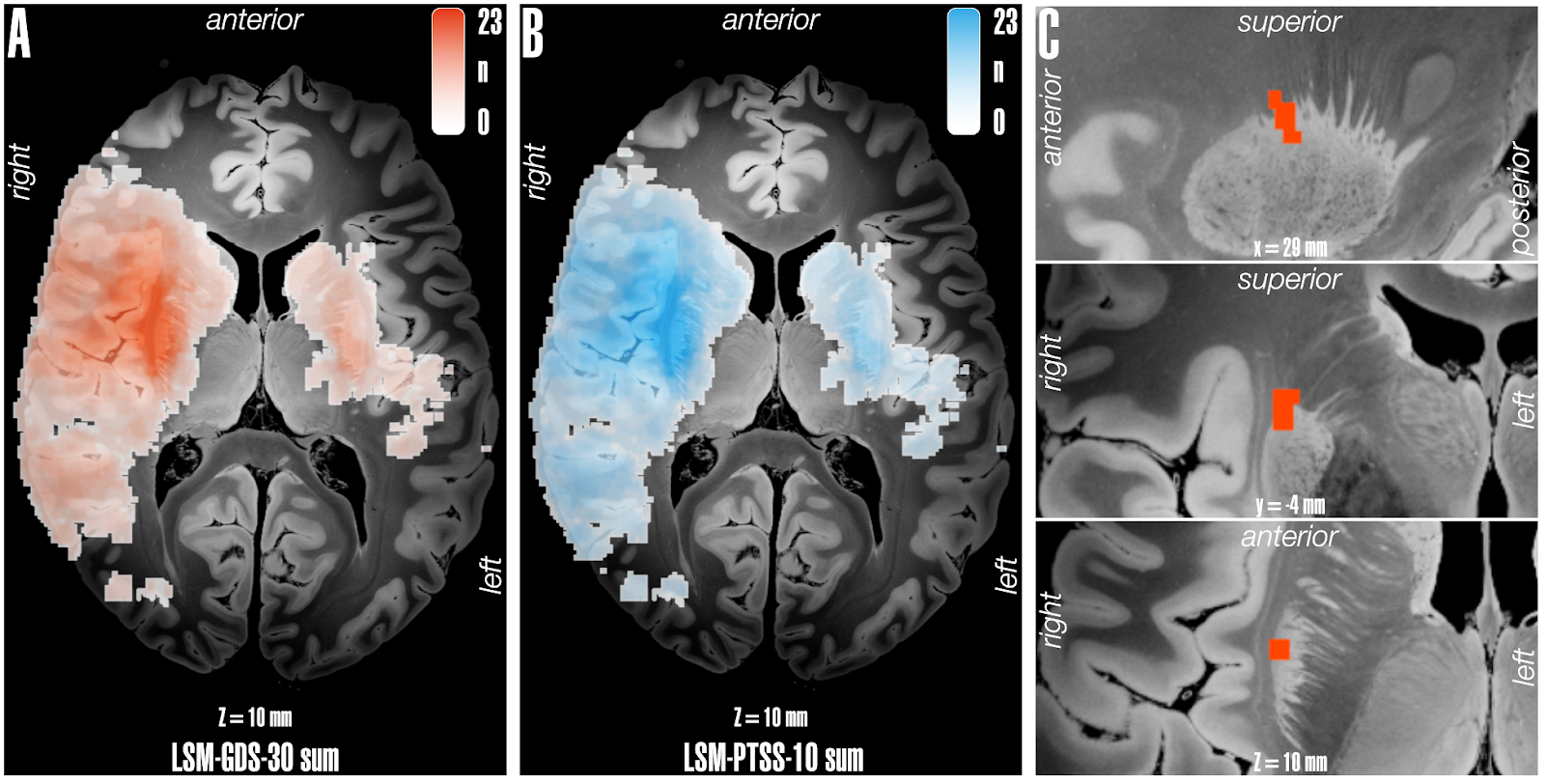
**Supplementary Figure 1:** Voxel-wise lesion symptom mapping. Sum of lesions included for VLSM analysis of A) depression and B) PTSD 12 months after stroke, in both analyses, voxels with a minimum overlap of 3 were included (n=23). No voxels survived significance threshold (FWE-p<0.050) in either whole-brain LSM or ROI-based LSM analyses for both outcomes of interest.

| **Linear regression analysis for GDS-30 at T3** | | | | |
| --- | --- | --- | --- | --- |
|  | **Univariable model** | | **Multivariable model** | |
|  | **Beta coefficient (95% CI)** | **p-value** | **Beta coefficient (95% CI)** | **p-value** |
| Striatal infarction | 1.6 (-0.28-3.5) | 0.094 | 1.7 (-0.19-3.7) | 0.076 |
| Age | -0.04 (-0.12-0.03) | 0.263 | -0.05 (-0.5 – 2.4) | 0.628 |
| Female | .19 (-0.17-2.1) | 0.837 | 0.47 (-0.19 – 3.7) | 0.628 |
| Lesion volume (mL) | 0.008 (-0.03-4.3) | 0.664 | 0.01 (-0.02 – 0.05) | 0.491 |
| **Linear regression analysis for PTSS-10 at T3** | | | | |
|  | **Univariable model** | | **Multivariable model** | |
|  | **Beta coefficient (95% CI)** | **p-value** | **Beta coefficient (95% CI)** | **p-value** |
| Striatal infarction | 1.6 (-2.2 – 5.4) | 0.405 | 1.8 (-1.9 – 5.5) | 0.352 |
| Age | -0.09 (-0.24 – 0.05) | 0.209 | -0.14 (-0.29 – 0.001) | 0.067 |
| Female | 4.1 (0.43-7.7) | 0.029 | 5.1 (1.3 – 8.8) | *0.009* |
| Lesion volume (mL) | 0.02 (-0.05 – 0.09) | 0.567 | 0.04 (-0.4 – 0.11) | 0.319 |

**Supplementary Table 3:** Univariable and multivariable linear regression models for GDS-30 and PTSS-10 total scores assessed 90 days post-stroke (T3), presenting crude and adjusted beta coefficients with 95% confidence intervals (CI).

| **Linear regression analysis for GDS-30 at T3** | | | |
| --- | --- | --- | --- |
|  | **Multivariable model** | |  |
|  | **Beta coefficient (95% CI)** | **p-value** |  |
| Striatal infarction | 1.5 (-0.39- 3.5) | 0.117 |  |
| Age | -0.05 (-0.12 – 0.03) | 0.221 |  |
| Female | 0.32 (-1.6 – 2.2) | 0.741 |  |
| Lesion volume (mL) | 0.01 (-0.02 – 0.05) | 0.509 |  |
| Antidepressants at any time (binary) | 2.7 (-0.58 – 6.1) | 0.104 |  |
| **Linear regression analysis for PTSS-10 at T3** | | | |
|  | **Multivariable model** | |  |
|  | **Beta coefficient (95% CI)** | **p-value** |  |
| Striatal infarction | 1.3 (-2.4 – 5.1) | 0.476 |  |
| Age | -0.14 (-0.28 – 0.001) | 0.065 |  |
| Female | 4.8 (1.0 – 8.4) | *0.013* |  |
| Lesion volume (mL) | 0.03 (-0.04 – 0.11) | 0.331 |  |
| Antidepressants at any time (binary) | 5.5 (-0.94 – 11.8) | 0.094 |  |

**Supplementary Table 4: Sensitivity analysis including medication status as an additional covariate.** Multivariable regression analysis for GDS-30 and PTSS-10 total score (continuous score) assessed 90 days post-stroke (T3) presenting crude and adjusted beta coefficients with 95% confidence intervals (CI).

| **Dependent variable: Geriatric Depression Score** | | | |  |
| --- | --- | --- | --- | --- |
| **Fixed-effects** | | | | |
|  | **Coefficient** | **Std. Error** | **p-value** | **95% CI** |
| Time-point of assessment  T2 (1d)  T3 (90d)  T4 (12mo) | *- reference -*  0.90  2.8 | *- reference -*  0.58  0.60 | -  0.115  *<0.001* | -0.19 – 2.0  1.6 – 3.9 |
| Age | -0.019 | 0.036 | 0.610 | -0.08 – 0.05 |
| Female | 0.70 | 0.95 | 0.455 | -1.2 – 2.4 |
| Lesion volume (mL) | 0.010 | 0.021 | 0.619 | -0.04 – 0.04 |
| Striatal network damage score | -0.24 | 0.6 | 0.696 | -0.62 – 1.2 |
| **Random-effects** | | | | |
|  | **Estimate** | **Std. Error** | |  |
| Subject ID | 3.2 | 0.38 | | 2.5 – 4.0 |
| **Dependent variable: Post-Traumatic Stress Syndrome 10-Question Inventory** | | | |  |
| **Fixed-effects** | | | | |
|  | **Coefficient** | **Std. Error** | **p-value** | **95% CI** |
| Time-point of assessment  T3 (90d)  T4 (12mo) | *- reference -*  0.93 | *- reference -*  1.1 | -  0.394 | -1.2 – 3.0 |
| Age | -0.18 | 0.08 | 0.025 | -0.3 - -0.02 |
| Female | 5.5 | 2.1 | 0.008 | 1.3 – 9.2 |
| Lesion volume (mL) | 0.06 | 0.05 | 0.158 | -0.04 – 0.13 |
| Striatal network damage score | -2.8 | 2.2 | 0.208 | -3.4 – 0.55 |
| **Random-Effects** | | | | |
|  | **Estimate** | **Std. Error** | |  |
| Subject ID | 6.4 | 0.55 | | 5.4 – 7.6 |

**Supplementary Table 5**: Linear Mixed-models for each primary outcome variable GDS-30 assessed at T2, T3, and T4 and PTSS-10 (assessed at T3 and T4). All models included subjects as a random effect and striatal network damage score, time point of assessment, female sex, age, and lesion volume in millilitres (mL).
